# In Vitro Effects of External Pressure Changes on the Sealing Ability under Simulated Diving Conditions

**DOI:** 10.5402/2012/418609

**Published:** 2012-10-15

**Authors:** Marcus Stoetzer, Martin Ruecker, Andreas Koch, Dirk Ziebolz, Horst Kokemüller, Christina Kaempf, Nils-Claudius Gellrich, Constantin von See

**Affiliations:** ^1^Department of Oral and Maxillofacial Surgery, Hanover Medical School, 30625 Hannover, Germany; ^2^Naval Institute of Maritime Medicine, Kiel, Germany; ^3^Department of Preventive Dentistry, Periodontology and Cariology, Medical Center Göttingen, Göttingen, Germany

## Abstract

*Aim*. To measure and validate the permeability of pressure changes in correlation to different root filling techniques. *Methods*. Eighty extracted single-rooted teeth were randomly assigned to one of eight groups of ten teeth. Following standardized instrumentation and irrigation, root canal fillings were performed using either cold lateral condensation, a warm carrier-based gutta-percha obturation technique, a warm carrier-based Resilon, or warm gutta-percha compaction with the downpack/backfill technique. After insertion of a pressure sensor within the pulp chamber ten teeth of each group then underwent simulated dives with pressure measurement and the other ten a dye penetration test during simulated dives to 5.0 bar. Differences were analyzed statistically (*P* < 0.05) using one-way analysis of variance (ANOVA). *Results*. When the warm carrier-based gutta-percha obturation technique and vertical gutta-percha obturation techniques were used, there was significant lower intrapulpal pressure to experimental chamber pressure (*P* > 0.05). When cold lateral condensation or carrier-based Resilon as used, pressure was sometimes almost completely equalized. *Conclusions*. Warm gutta-percha obturation techniques provide a largely pressure-tight seal whereas the Resilon obturation technique and cold lateral condensation appear to be unsuitable to pressure changes.

## 1. Introduction

Root canal fillings must meet a number of requirements. Not only must they be biocompatible and removable, but they must also tightly seal the root canal system. The effectiveness of the seal depends on the anatomy of the root canal system and especially on the shape of the canal and the type of mechanical preparation. It is undisputed that an effective seal can be technically achieved if the root canal is tapered from crown to apex [1]. Different materials and methods can be used to fill and seal a root canal. Differences mainly pertain to how filling materials are processed and whether accessory ramifications can be filled effectively [2]. Cold lateral condensation is still a widely accepted method of root canal obturation, although accessory ramifications can hardly be filled using this technique [3]. Apart from this technique, warm thermoplastic obturation methods in particular have become established in recent years [4]. These techniques use either carrier-based obturation material or simply heated filling material [5].

Many studies are available that have investigated the adaptation of root fillings to the root canal walls and the ability of filling materials to seal the root canal system. For analysis of the adaptation dye penetration tests, which are simple to perform and relatively easy to evaluate, are available for this purpose [6]. Although a comparison of different techniques in one study is usually possible, it is often difficult to compare the results of studies because different and nonstandardized methods were used [7]. In many cases, studies were conducted in different settings. Ambient conditions are thus likely to have a notable influence on the sealing ability of root canal fillings. No studies, however, have so far been conducted under extreme ambient conditions, for example at the extremely high pressure levels associated with diving. Depending on diving depth, different physiological and pathological processes take place in the human body [8]. Barotrauma is defined as pressure-induced damage and can occur both at high and low pressures. The pathology of barotrauma is directly related to Boyle's law, which states that if temperature remains constant, the volume of a fixed mass of an ideal gas is inversely proportional to the pressure of the gas. As pressure increases, the volume of a confined gas decreases. Vice versa, volume increases as pressure decreases. Pressure differences (Table 1) can occur in the human body when a gas-filled cavity cannot communicate with the exterior and pressure cannot be equalized. Clinically, the resulting pressure difference between the gas-filled cavity and the exterior environment can lead to pain, edema, or vascular gas embolism [9]. Tooth pain occurring with changes in pressure is generally referred to as barodontalgia [10]. Since the etiology of barodontalgia is still not completely understood, current dental treatment recommendations for flying and diving personnel are often based on statistical data [11]. In the literature, there are no studies addressing pressure and fluid shifts along root canal fillings in association with external pressure changes. The objective of this barometric study was therefore to investigate the relationship between external pressure and intrapulpal pressure and to assess the influence of external pressure changes on the sealing ability of different types of root canal fillings and obturation techniques.

## 2. Material and Methods

### 2.1. Study Design

Eighty extracted single-rooted human teeth (premolar) were randomly assigned to one of eight groups, each consisting of ten teeth. None of the teeth showed signs of dental caries or had been treated endodontically. Only teeth with a root canal curvature of less than 20 degrees were included in the study. 

Following standardized machine instrumentation to a minimum preparation size of ISO # 30.04 (VDW Gold, VDW, Munich, Germany) and irrigation with 2.25% sodium hypochlorite (NaOCl) activated by ultrasound and 0.2% chlorhexidine (CHX), the root canals were dried with paper points. A light coating of AH Plus sealer (AH Plus, Detrey, Konstanz, Germany) was then applied to the root canal. The canals were filled to a depth 1 mm short of the apical foramen using either cold lateral condensation (Table 2) (*n* = 20), a warm carrier-based gutta-percha obturation technique (Guttamaster, VDW, Germany) (*n* = 20), a warm carrier-based Resilon obturation technique (Sybronendo, CA, United States) (*n* = 20), or a warm gutta-percha compaction with the downpack/backfill technique (BeeFill, VDW, Munich, Germany) (*n* = 20). A single endodontist with special training in the respective filling techniques performed all root canal preparations and obturations in a standardized manner in order to minimize procedural variations. The length and homogeneity of the root canal fillings was assessed using dental radiographs, which were taken in two planes. Prior to the measurements, the teeth were stored for 24 hours at 37°C and a humidity of 90%. 

Ten teeth of each group underwent intrapulpal pressure measurement and the other ten teeth a dye penetration test during simulated dives to an external pressure of 5.0 bar. Pressure differences in the pulp cavity and dye penetration depths from the apex were measured.

### 2.2. Intrapulpal Pressure Measurement

For pressure measurement the manometer at the diving chamber and the pressure sensor were calibrated against each other prior to the first measurement. The pressure inside the chamber was directly read from the manometer at the chamber. The pressure within the pulp cavity was measured at a frequency of 0.5 Hz using computer software (Measure Foundry Version 4.0, Data Translation, Inc., Marlboro, United States).

The difference between the pressure in the chamber and the pressure in the pulp cavity was calculated at predefined time points for every 0.5 bar using the formula Δ*p* = *p*_*C*_ − *p*_*P*_, where *p*_*C*_ is the pressure inside the chamber and *p*_*P*_ is the pressure in the pulp cavity. 

### 2.3. Experimental Protocol

The simulated dives were performed in a specially equipped diving chamber (Haux, Draegerwerk, Luebeck, Germany) using compressed air. Inlet valves allowed us to variably adjust the pressure increase inside the chamber. A manometer indicated the pressure in the chamber. 

For the measurement of pressure inside the pulp cavity, a circular hole with a diameter of 6.0 mm was drilled from the crown through the enamel and dentine to the pulp chamber using a diamond burr (S-5 mm, Saint-Gobain, Norderstedt, Germany) with constant water cooling. A pressure sensor (4005BA5, Kistler, Ostfildern, Germany) was inserted into the cavity and the measuring sensor was placed in the roof of the pulp cavity. The gap between the tooth and the threads of the pressure sensor was then tightly sealed using an acid etch technique and a low-viscosity dental composite. Finally, impression material (KNET NF, Pluradent, Offenbach, Germany) was used to fix the tooth in such a way that the entire surface of the root was covered with a 0.5-mm-thick layer of water from the root apex to the neck of the tooth. 

### 2.4. Dye Penetration Test

In order to prepare the teeth for the dye penetration test with methylene blue, we applied two layers of white nail varnish to the teeth except for the apical 2 mm, which remained exposed. The teeth were inserted into a plastic container filled with 2% methylene blue in such a way that they were completely covered with dye. The container was then placed into the experimental pressure chamber. 

A dive was simulated and consisted of a descent to 5.0 bar in 2.5 minutes. The pressure was maintained for two hours and the subsequent return to ambient pressure was undertaken again over a period of 2.5 minutes.

The teeth were cleaned under running water. Residual dye was removed and a scaler was used to remove nail varnish. After the teeth were decalcified and made transparent according to a standard procedure, the depth of penetration was measured from four sides under a microscope at 20x magnification. The measured values were averaged. 

### 2.5. Statistical Analysis

The normal distribution and homogeneity of variance were assessed. Results are expressed as means ± SEM. Differences between pressure chamber and pulp cavity pressure were evaluated with a one-way analysis of variance (ANOVA). A *P* value < 0.05 was considered significant.

## 3. Results

### 3.1. Intrapulpal Pressure Measurement

Intrapulpal pressure measurements revealed considerable differences both between and within the various groups of teeth in the ability of the root fillings to achieve a hermetic seal.

When the cold lateral condensation technique was used, pressure in the chamber and pressure inside the pulp cavity were completely equalized in two teeth. A minor intrapulpal pressure increase was observed in five teeth. No pressure equalization occurred in the three remaining teeth. 

When the warm carrier-based gutta-percha obturation technique was used, a minor intrapulpal pressure increase by not more than 0.2 bar was observed in only one tooth. Equalization of pressure did not occur in any of these teeth at 5.0 bar chamber pressure. 

By contrast, almost complete pressure equalization took place in half of the teeth when the carrier-based Resilon obturation technique was used. Minor pressure increases ranging between 0.3 and 0.7 bar were observed in the other teeth of this group. 

When root canals were filled with warm vertical gutta-percha (Downpack/backfill), there was no significant change in intrapulpal pressure in response to an increase in external pressure.

### 3.2. Dye Penetration Test

The cold lateral condensation technique showed moderate dye penetration from the apex. 

When the warm carrier-based gutta-percha obturation technique was used only minor dye penetration was observed. By contrast, significantly deeper dye penetration was observed for the warm carrier-based Resilon obturation technique. Half of the examined teeth showed total dye penetration in the entire root canal system. The warm vertical and warm carrier-based gutta-percha obturation techniques showed the least dye penetration.

## 4. Discussion

The obturation techniques that we studied in vitro showed significant differences in their ability to hermetically seal the root canal system. A safe hermetic seal was achieved only by warm carrier-based gutta-percha obturation technique and warm vertical gutta-percha compaction. 

The quality of root fillings can be evaluated with a variety of methods such as microscopic evaluations, cross-sections, fluid transport tests, and dye penetration tests. In the study presented here, we used methylene blue in order to study marginal infiltration and to facilitate the comparison of our results with other studies. In addition, we used transparent teeth rather than cross-sections in order to be able to evaluate the teeth without further manipulation. Although a dye penetration test is a widely used and convenient technique for assessing leakage in vitro, it is still unclear whether the results reflect microbial leakage in vivo [12].

Furthermore, we used a piezo-sensor to measure intrapulpal pressure. This method allowed us to assess the hermetic sealing of root fillings and to assess the effectiveness of the seal at changing external pressure levels. We were thus able to control external pressure and internal pulp cavity pressure changes. In our tests, we increased pressure by 2.0 bar per minute in order to simulate an ascent without decompression stops. This method of pressure measurement, however, allowed us to assess the hermetic sealing of the entire root canal system but not to localize possible sites of permeability. These sites require additional tests such as a dye penetration test. 

Simulated root canals in plastic blocks or extracted natural teeth have proved to be useful in systematically examining root fillings in vitro. In this context, natural teeth are superior to plastic blocks since they better reflect the dentin surface and the resulting mechanical properties at the interface between dentin and root filling material. Depending on the configuration of the root canal, natural teeth differ widely in the anatomy of the root canal system. In addition, root curvature plays an especially important role in the preparation and obturation of the root canal system. In our study, we therefore used only single-root extracted teeth with a root canal curvature of less than 20 degrees in order to facilitate comparability. 

The root canals were prepared with nickel-titanium (Ni-Ti) files since these instruments can produce canals that are more uniform, better centered, and rounder than those created by hand files. In addition, we finally used ultrasonic irrigation to remove debris and the smear layer and thus to increase the adaptation of the fillings to the root canal walls [13].

One of the main objectives of root canal treatment is to completely seal the root canal system and thus to prevent bacterial recontamination. For this purpose, sealers should be used in order to achieve as effective a seal as possible [14]. Sealers differ in their cytological and mechanical properties and especially in their chemical composition, which determines dimension stability and thus influences the leakage behavior of root canal fillings [15, 16]. In our tests, we used AH Plus, which is available in a syringe that automatically mixes the sealer components and thus minimizes the risk of air entrapment during mixing. Compared with other sealers, AH Plus was found to provide the most effective hermetic seal under standard conditions [17]. Our tests do not allow reliable conclusions to be drawn about the behavior of sealers when exposed to changes in atmospheric pressure. Since we used the same sealer and the same method of application in all tests, between-group comparisons are, however, possible and reasonable. 

A variety of systems are available for obturating root canals [18]. Gutta-percha is the most widely used filling material and is still regarded as the gold standard. It is suitable for both cold and warm obturation techniques. In the literature, studies comparing cold and warm methods of obturation in terms of leakage under standard conditions are not uniform. Cold lateral condensation is one of the most widely used obturation techniques. Compared with thermoplastic obturation techniques, cold lateral condensation is reported to have possible disadvantages such as inhomogeneity, an increased risk of canal fracture, and poor adaptation to the canal walls [19]. Our study demonstrated a wide variance in the cold lateral condensation group during the pressure tests. Likewise, dye penetration tests showed a wide variance in the depth of penetration. Possible explanations are a potentially increased proportion of sealer in the root filling or a poor marginal seal under higher pressure levels. 

When the root canals were filled with Resilon, pressure in the chamber and pressure in the pulp cavity were completely equalized in half of the teeth. Since a warm carrier-based technique was used to insert Resilon, inhomogeneity of the root canal filling, which can be observed after cold lateral condensation, is improbable. It is more likely that there was no hermetic seal between filling and canal wall. For this reason, it appears doubtful that a pressure-tight seal can be achieved when the combination of AH Plus and Resilon is used. This is supported by the results published by Onay et al. and Pasqualini et al., who reported that the combination of Resilon and AH Plus exhibited greater leakage than a combination of sealer and gutta-percha [20]. In addition, De-Deus et al. were able to show an increased permeability of Resilon fillings after stress exposure [21]. An increase in external pressure is a type of mechanical stress and our results thus add to existing knowledge about the resistance of Resilon to stress [22, 23].

By contrast, obturation with warm gutta-percha showed better results in both the pressure measurements and the dye penetration tests irrespective of the method of application. When the warm carrier-based gutta-percha obturation technique was used, a minor intrapulpal pressure increase of no more than 0.2 bar was observed. When warm vertical compaction was used, there was no pressure increase at all. In the dye penetration test, warm vertical compaction showed results similar to those obtained for the warm carrier-based obturation technique with less variance. These findings are in line with the results by Lea et al., who reported that warm gutta-percha obturation techniques achieved a better seal than cold lateral compaction [24]. 

In conclusion, warm gutta-percha obturation techniques should be preferred to cold lateral condensation or warm carrier-based Resilon obturation techniques in the endodontic treatment of patients such as professional divers or parachutists who are often exposed to changes in atmospheric pressure.

## Figures and Tables

**Table 1:** Pressure differences of the pressure chamber and the pulp cavity for different root filling techniques at 5.0 bar chamber pressure (significance *P < 0.05 versus cold lateral condensation).

Group		Δ*p* [bar]	SEM
Cold lateral condensation	*n* = 10	1.33	0.9
Carrier-based gutta-percha obturation	*n* = 10	0.09	0.04
Carrier-based Resilon obturation	*n* = 10	1.94	1.32
Downpack/backfill (Gutta-percha)	*n* = 10	0.03	0.02

**Table 2:** Apical dye leakage penetration after simulated diving up to 5.0 bar for different root filling techniques.

Group		Mean [mm]	SEM
Cold lateral condensation	*n* = 10	2.36	3.23
Carrier-based gutta-percha obturation	*n* = 10	1.32	1.39
Carrier-based Resilon obturation	*n* = 10	8.05	3.85
Downpack/backfill (Gutta-percha)	*n* = 10	1.29	0.65
